# A Protocol for Studying HIV-1 Envelope Glycoprotein Function

**DOI:** 10.1016/j.xpro.2020.100133

**Published:** 2020-10-14

**Authors:** Sneha Ratnapriya, Angela Chov, Alon Herschhorn

**Affiliations:** 1 Division of Infectious Diseases and International Medicine, Department of Medicine, University of Minnesota, Minneapolis, MN 55455, USA; 2 Microbiology, Immunology, and Cancer Biology Graduate Program, University of Minnesota, Minneapolis, MN 55455, USA; 3 The College of Veterinary Medicine Graduate Program, University of Minnesota, Minneapolis, MN 55455, USA; 4 Institute for Molecular Virology, University of Minnesota, Minneapolis, MN 55455, USA

## Abstract

HIV-1 envelope glycoproteins (Envs) bind to CD4 receptor and CCR5/CXCR4 coreceptor and mediate viral entry (Feng et al., 1996; Herschhorn et al., 2016, 2017; Kwong et al., 1998). HIV-1 Envs are the sole target of neutralizing antibodies and a main focus of vaccine development (Flemming et al., 2018). Here, we provide a step-by-step protocol to measure Env sensitivity to ligands, cold, and small molecules, as well as to study viral infectivity and to dissect parameters affecting HIV-1 Env function.

For complete details on the use and execution of this protocol, please refer to Harris et al. (2020).

## Highlights

• Pseudoviruses are used to display and study HIV-1 envelope glycoproteins (Envs)
• We describe the steps to measure HIV-1 sensitivity to Env ligands and cold
• Cell-cell fusion activity is critical for HIV-1 Env function

## Graphical Abstract

**Figure:**
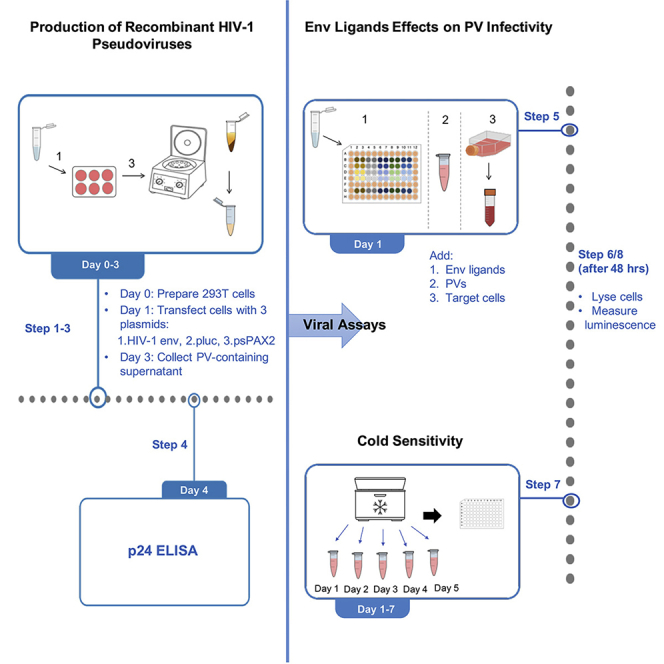

## Before You Begin

### Biosafety

The National Institutes of Health (NIH) guidelines classify HIV-1 as a Risk Group 3 agent. Any research involving HIV-1-based lentiviral vectors should be approved by the institutional biosafety committee and carried out according to the recombinant DNA advisory committee (RAC) guidance. Additional details can be found in the following document:

https://osp.od.nih.gov/wp-content/uploads/Lenti_Containment_Guidance.pdf

We routinely use single round pseudoviruses (PVs) carrying a reporter gene to study HIV-1 envelope glycoprotein function. We prepare the PVs by co-transfecting cells with (1) a lentiviral vector that provides HIV-1 structural proteins and enzymes, (2) a firefly luciferase reporter vector, and (3) an envelope-expressing plasmid. Upon infection, the expression level of the Luciferase reporter protein in the infected cells gives an estimate of the extent of PVs infection. The flexibility of the system allows to combine the same core plasmids (1 and 2) with different viral envelope plasmids of interest. Thus, the system allows rapid and precise measurements of specific viral envelope function.

### Cell Maintenance

**Timing: 2–3 days**

We use healthy, exponentially growing 293T cells for the production of HIV-based pseudoviruses (PVs)—viruses displaying an envelope of a different origin or strain—and TZM-bl or Cf2Th-CD4/CCR5 cells for measuring PV infectivity. Cells can be stored for years in the vapor phase of a liquid nitrogen storage tank.

**Figure 1:**
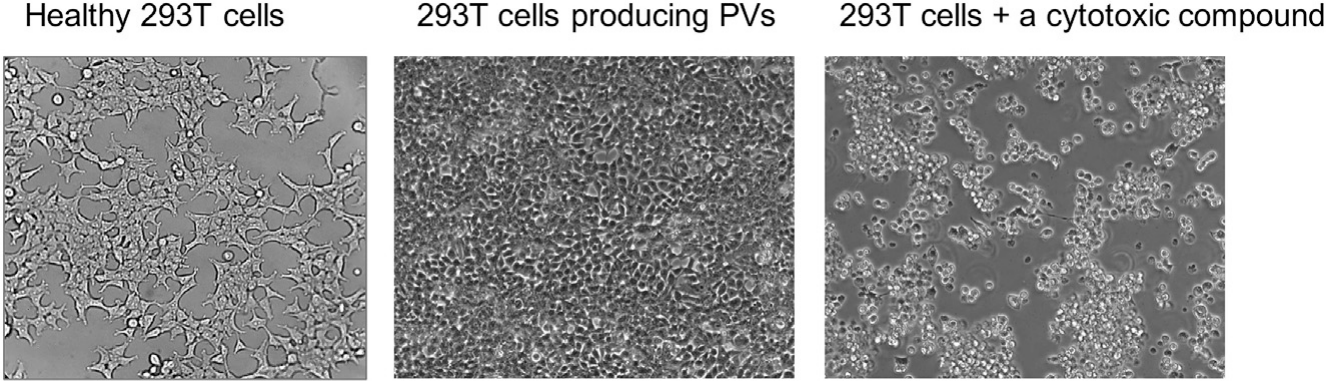
293T Cells Cells that are healthy or under different experimental conditions are shown.

1. Thaw the cells quickly at 37°C by immersing the tube in a water bath.
2. Maintain 293T cells (Figure 1) in Dulbecco’s Modified Eagle Medium (DMEM) containing 2 mM glutamine, 4.5 g/L glucose, 10% FBS, 100 μg/mL streptomycin and 100 units/mL penicillin.

3. Maintain TZM-bl cells in DMEM containing 10% FBS, 100 μg/mL streptomycin and 100 units/mL penicillin.
4. Maintain Cf2Th-CD4/CCR5 cells in DMEM containing 10% FBS, 100 μg/mL streptomycin and 100 units/mL penicillin, supplemented with 400 μg/mL G418 and 200 μg/mL hygromycin B antibiotics.

***Note:*** All cells should be passaged at least twice and should show healthy morphology before using them in the viral assays. We have not seen significant decrease in transfection efficiency (293T) or reporter activity (TZM-bl or Cf2Th-CD4/CCR5) even after 30 passages. We typically split the cells at <90% confluency (every 2–3 days) using the following protocol: (1) Remove the media from the flask, followed by washing once with PBS. (2) Add 1–2 mL of dissociation reagent (TrypLE or StemPro Accutase) to the adhered cells and incubate until cells are detached (typically < 5 min). (3) Add 10 mL of DMEM to the flask, collect the detached cells, and mix slowly by gentle pipetting up and down. (4) Dilute the cell suspension as necessary and transfer the required volume of cells to a new flask. All cells are typically maintained in vented culture flasks at 37°C, incubated with 5% CO_2_.

### Prepare Plasmids for Transfection

**Timing: 3 days**

5. Prepare three plasmids listed below (all contain an ampicillin-resistant gene (*beta-lactamase*)).
  a. An envelope-expression plasmid (e.g., pCDNA-based expression plasmid).
  b. A lentiviral reporter vector containing the *firefly luciferase* gene (e.g., pHIVec2.luc).
  c. HIV-1-based packaging plasmid (e.g., psPAX2).
6. Plasmid DNA stocks are prepared by transformation of original plasmids into chemically competent DH5a or Stbl3 *E Coli* bacteria by standard heat shock protocol.

***Alternatives:*** Mix & Go *E. coli* transformation kit (T3001; Zymo Research) can be used to avoid the need for heat shock, incubations, or outgrowth steps.

7. Select from the transformed bacteria plate a well separated and rounded single bacteria colony.
8. Inoculate the bacteria in 2 mL Luria Bertani (LB) broth supplemented with 100 μg/mL ampicillin or carbenicillin (a stable derivative of ampicillin).
9. Grow the transformed bacteria for 16 h in a shaker incubator at 37°C for small scale minipreps or dilute them after 6–8 h to 250 mL of the same LB medium containing antibiotics and allow the bacteria to grow overnight (16–20 h) for medium scale midipreps.

***Note:*** Several commercial miniprep and midiprep kits are available from different vendors to purify plasmid DNA. Follow the manufacturer’s instructions.

**CRITICAL:** Transformed pHIVec2.luc plasmid is maintained in *E. Coli* bacteria at a low-copy number and therefore the pHIVec2.luc plasmid should be prepared from a large volume culture. We usually grow the transformed bacteria in two to three 1-liter flasks containing bacteria in 250 mL LB broth each.

## Key Resources Table

**Table:** 

REAGENT or RESOURCE	SOURCE	IDENTIFIER
**Chemicals, Peptides, and Recombinant Proteins**		

Adenosine 5′-Triphosphate Disodium salt Hydrate (ATP)	Sigma	Cat# A26209-10G
Agar	BD	Cat# 281230
Ampicillin Sodium Salt	Sigma	Cat# A9518-5G
Bovine Serum Albumin (BSA)	Sigma	Cat# A2153-100G
Calcium Chloride (CaCl_2_)	Sigma	Cat# C7902-500G
Carbenicillin Disodium Salt	VWR Life Science	Cat# J358-1G
Chloroquine	Sigma	Cat# C6628-25G
D-Luciferin Phosphate (chemical name: D-(-)-2-(6′-hydroxy-2′-benzothiazolyl)-thiazoline-4-carboxylic acid)	BD Biosciences	Cat# 556879
Dimethyl Sulfoxide (DMSO)	Sigma	Cat# D2438-10ML
Dithiothreitol (DTT)	Sigma	Cat# 43816-10ML
Dulbecco’s Modified Eagle Medium (DMEM)	Gibco	Cat# 11965-084
Dulbecco’s Phosphate Buffered Saline (PBS)	Sigma	Cat# D8537-500ML
Effectene Transfection Reagent	Qiagen	Cat# 301425
Ethylene Diamine Tetra Acetic Acid (EDTA)	Promega	Cat# V4231
Fetal Bovine Serum (FBS)	Gibco	Cat# 10437-010
Geneticin G418 Sulfate	Invitrogen	Cat# 10131027
Glucose	Alfa Aesar	Cat# A16828
Glycerol	Fisher Chemical	Cat# G33-500
HEPES	Sigma	Cat# H4034-25G
Hydrochloric Acid (HCl)	Ricca Chemical Company	Cat# 3700-16
Hydrogen Peroxide Solution (30% w/w)	Sigma	H1009-100ML
Hygromycin B	Invitrogen	Cat# 10687010
Kanamycin	VWR Life Science	Cat# 0408-25G
Magnesium Sulfate (MgSO_4_)	Sigma	M1880-500G
Penicillin-Streptomycin (PenStrep)	Gibco	Cat# 15140-122
Phosphoric Acid (H_3_PO_4_)	Sigma	Cat# 466123-25G
Potassium Chloride (KCl)	Sigma	Cat# P5405-250G
Potassium Phosphate Dibasic (K_2_HPO_4_)	Sigma	795496-500G
Potassium Phosphate Monobasic (KH_2_PO_4_)	Sigma	795488-500G
Sodium Chloride (NaCl)	Sigma	Cat# S5886-5KG
Sodium Hydroxide (NaOH)	Sigma	Cat# 58045-500G
Sodium Phosphate Dibasic (Na_2_HPO_4_)	Sigma	Cat# S5136-100G
StemPro Accutase	Gibco	Cat# A11105-01
Sulfuric Acid (H_2_SO_4_) 2.0 N (1.0 M)	LabChem	Cat# LC257901
3,3′,5,5′-Tetramethylbenzidine (TMB)	Sigma	Cat# T2885-5G
Trans-1,2-Diaminocyclohexane-N,N,N′,N′-tetra acetic acid monohydrate (DCTA)	Sigma	Cat# 319945-25G
Tris Base	Fisher BioReagents	Cat# BP152-1
Triton-X 100	Sigma	Cat# X100-100ML
TrypLE Express (-) phenol red	Gibco	Cat# 12604-021
Tryptone	BD	Cat# 211705
Tween-20	BIO-RAD	170-6531
Yeast Extract	BD	Cat# 212750

**Critical Commercial Assays**		

HIV-1 p24 antigen capture assay	Advanced BioScience Laboratories	Cat# 5421
p24 Duo ELISA set	R&D Systems	Cat# DY7360-05

**Experimental Models: Cell Lines**		

Cf2Th CD4/CCR5 cells	Laboratory of Joseph Sodroski	Parental Cf2Th cells are from ATCC (CRL-1430)
HEK 293T/17 cells	ATCC	CRL-11268
TZM-bl cells	NIH AIDS Reagent Program	Cat# 8129

**Recombinant DNA**		

pCO-HIV-1_JRFL_ env	Herschhorn lab	Env-expression plasmid
pCO-HIV-2_UC1_ env	Herschhorn lab	Env-expression plasmid
psPAX2	NIH AIDS Reagent Program	Cat# 11348
pHIVec2.luc	Laboratory of Joseph Sodroski	Based on pHIVec2.gfp (Hofmann et al., 1999)
pCO-HIV-1_JRFL_ gp120	Current study	pcDNA™3.1/Zeo ^(-)^ backbone plasmid (Invitrogen)
pCO-sCD4	Current study	pcDNA 3-based backbone plasmid (Invitrogen)

**Software and Algorithms**		

Gen5	BioTek Instruments	Version 2.09
MikroWin 2000 Lite	Berthold Technologies GmbH	Id. Nr. 37854-304

**Other**		

Cell Culture Microplate 96-well, PS, F-Bottom, White, Lid with condensation rings, Sterile (Luminometer plates)	Greiner bio-one	Cat# 655083
Microplate luminometer	Berthold Technologies GmbH	Centro LB 960 XS^3^
Spectrophotometer	BioTek	SYNERGY/H1 microplate reader
∗Central vacuum line	N/A	N/A
∗Collection Flasks (1L conical Pyrex flasks)	Corning	Cat# 5340
∗0.22 μm filter (50 mm)	Membrane Solutions	Cat# SFPTFE050045SBH
∗Rubber stopper	MilliporeSigma	Cat# 164380-16EA
∗Rubber clear tygon tubing	W.W. Grainger	Cat# R-3603
∗8-tip manifold	MilliporeSigma Mansion Labs	Cat# BR704526-1EA Cat# 0166-704526

**Figure 2:**
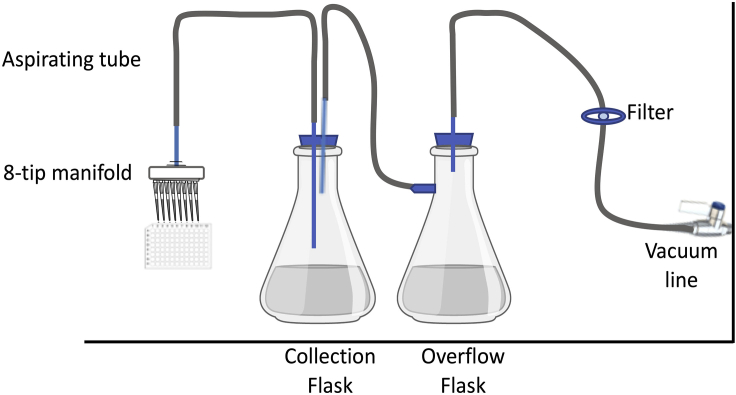
An In-House Vacuum System Setup Different components of the in-house vacuum system are shown.

∗Components for the in-house vacuum system (Figure 2).

## Materials and Equipment

### Transfections

Different transfection reagents are commercially available or can be prepared in the laboratory. Here we describe two alternatives: (1) calcium phosphate [Ca_3_(PO_4_)_2_], and (2) Effectene reagent (Qiagen).

• **Calcium phosphate.** Efficiency of Ca_3_(PO_4_)_2_-mediated transfection depends on the exact pH of the transfection mix. We usually prepare three solutions of 2× HEPES buffer with pH values ranging from 6.8 to 7.2 and empirically test the transfection efficiency of each solution.
  ○ Prepare the following 2× HEPES buffer:

**Table:** 

Reagent	Final Concentration	Stock Concentration	Amount	Volume
HEPES	50 mM	-	1429.9 mg	-
KCl	10 mM	100 mM	-	12 mL
Glucose	12 mM	120 mM	-	12 mL
NaCl	280 mM	-	1963.6 mg	-
Na_2_HPO_4_	1.5 mM	150 mM	-	1.2 mL
ddH_2_O	-	-	-	Up to 105 mL

Split the 2× HEPES buffer to three beakers, 35 mL in each, and titrate the three solutions to pH 6.8, pH 7.05, and pH 7.2 using 5M NaOH. Add ddH_2_O up to 40 mL to each beaker, filter-sterilize the three solutions using 0.22 μm syringe filter, aliquot in 1.5 mL microcentrifuge tubes and store at -20°C.

  ○ Prepare 2M calcium chloride in ddH_2_O solution. Aliquot the solution in 1.5 mL microcentrifuge tubes and store at -20°C.

***Alternatives:*** These solutions can be purchased commercially as a calcium phosphate transfection kit (e.g., K278001 from Invitrogen).

***Note:*** pH is critical for transfection; all three buffers at different pH values should be tested by transfection of a plasmid containing reporter gene (e.g., green fluorescent protein (gfp)) and measuring the expression level of the reporter protein or the efficiency of transfection. We routinely achieve >90% transfection efficiency with plasmid containing the gfp gene and optimal Ca_3_(PO_4_)_2_ reagents. Solutions are usually stable for only 1–2 months at -20°C.

***Note:*** Ca_3_(PO_4_)_2_ transfection is based on forming calcium phosphate-DNA precipitates that facilitate DNA entry into target cells. It is a quick, simple, and inexpensive method with high transfection efficiency of 293T cells. In our hands, Ca_3_(PO_4_)_2_-mediated transfection into 293T cells is as efficient as other commercially available reagents.

• **Effectene** is commercially available from Qiagen and is used according to manufacturer’s instructions. We typically observe >90% transfection efficiency of 293T cells using Effectene.

### p24 ELISA Solutions

• 10× Phosphate buffered saline (PBS) buffer:

**Table:** 

Reagent	Final Concentration (10X)	Amount	Volume
NaCl	1.37 M	80 g	-
KCl	27 mM	2 g	-
Na_2_HPO_4_	100 mM	14.4 g	-
KH_2_PO_4_	18 mM	2.4 g	-
ddH_2_O	-	-	Up to 950 mL

Adjust pH to 7.4 with HCl and add ddH_2_O up to a volume of 1000 mL. Store at room temperature (20°C–25°C).

• Wash buffer solution: 0.05% Tween-20 in PBS (pH 7.4)
• Blocking buffer solution: Prepare 1% BSA in PBS (pH 7.4), filter with 0.2 μm filter and add 0.2% Triton X-100
• Tetramethylbenzidine (TMB) substrate solution: 300 μL of 4 mg/mL TMB substrate (dissolved in DMSO; TMB stocks can be stored at −20°C); 10 mL sodium acetate (pH 5.0); 5 μL of 30% hydrogen peroxide

***Alternatives:*** TMB substrate can be purchased commercially (e.g., Pierce™ TMB Substrate Kit Cat# 34021).

• Stop solution:
2 N H_2_SO_4_

**CRITICAL:** H_2_SO_4_ is a highly corrosive chemical and should be handled in a chemical hood with gloves, eye glasses, and proper PPE.

**Table:** Luciferase Lysis Buffer

Reagent	Final Concentration	Stock Concentration	Amount	Volume
Tris	25 mM	-	0.6057 g	-
DCTA^a^	2mM	-	0.1457 g	-
Triton X-100	1% (v/v)	-	-	2 mL
Glycerol	10% (v/v)	-	-	20 mL
Dithiothreitol	2 mM	1 M	-	0.4 mL
ddH_2_O	-	-	-	Up to 150 mL

a Trans-1,2-Diaminocyclohexane-N,N,N′,N′-tetra acetic acid monohydrate

Titrate with 15% Phosphoric acid (H_3_PO_4_) to pH 7.8 and add ddH_2_O up to 200 mL. Store at 4^o^C.

**Table:** Firefly Luciferase Assay Buffer

Reagent	Final Concentration	Stock Concentration	Volume
MgSO_4_	15 mM	0.25 mM	1.2 mL
Phosphate Buffer (KH_2_PO_4/_K_2_HPO_4_) pH 7.8	15 mM	0.25mM	1.2 mL
ATP	1 mM	100 mM	0.2 mL
DTT	1 mM	1M	0.02 mL
ddH_2_O	-	-	17.38 mL

### Luciferin Substrate Solution

Reconstitute lyophilized Luciferin with ddH_2_O and adjust the pH to 6.0–6.3 with HCl/NaOH. Store in aliquots at –20°C. Work quickly and protect from light for any luciferin-based solutions.

***Alternatives:*** Luciferase assay system that does not require the use of a luminometer with injectors can be purchased commercially (e.g., Bright-Glo™ Luciferase Assay System from Promega).

### Spectrophotometer

A spectrophotometer microplate reader is required to measure optical density at the last step of the p24 ELISA in order to measure the concentration of HIV-1 p24 in PV preparations.

### Vacuum System

We installed an in-house vacuum system connected through central vacuum supply (Figure 2). In this setup, the central vacuum line is sequentially connected to a 0.22 μm filter and two collection flasks (traps) followed by an aspirating tube. A serological pipette is connected to the tube for focused aspirating. This vacuum system provides manual flow control and works efficiently for aspirating the liquid from cell culture plates during ELISA procedures or before adding the lysis buffer to 96-well assay plates.

### Luminometer

A luminometer is required to measure the reporter luciferase activity, which results in an extremely sensitive readout with a dynamic range of more than 7 orders of magnitude. Since luciferin readout decays exponentially with a very short half-life, a luminometer equipped with injectors is required to read each well after a predefined time window to ensure reproducible results. We use the Centro XS^3^ LB960 (Berthold) to measure the signal in our assay but any other injector-based luminometer should give comparable results. Alternatively, commercially available luciferin-based substrate that is stabilized to have a long half-life (typically a few hours) can be used without the need for injectors.

## Step-By-Step Method Details

### Production of Recombinant HIV-1 Pseudoviruses

**Timing: 1 week**

1. Day 0: Maintain 293T cells in DMEM medium and monitor their growth rate and morphology. Cells should grow exponentially with a doubling time of approximately 24 h and show healthy morphology without significant cell aggregation. Wash 293T cells once with PBS and add 1–2 mL of StemPro Accutase, which gently detaches the 293T cells while preserving the expression of the receptors on the cell surface. Incubate at RT (20°C–25°C) until cells are completely detached, add 10 mL of DMEM to the flask, collect the detached 293T cells, and mix slowly by pipetting up and down. The cell suspension can be filtered if they tend to aggregate. We usually use 5 mL polystyrene round-bottom tube with cell-strainer cap to filter the cell suspension.
2. Count the cells using a hemocytometer and add 5 × 10^5^ cells/well to a 6-well plate or 3 × 10^6^ cells/T-25 flask. Incubate the plate/flask overnight (14–20 h) in the tissue culture incubator at 37°C and 5% CO_2_ concentration.
3. Day 1: Effectene transfection.
  a. Replace the medium with a fresh DMEM medium and follow manufacturer’s instructions. A typical template for preparing the transfection mixture for a 6-well plate transfection is shown in Table 1. Add dropwise the transfection mixture to the cells with gentle swirling.

  b. Gently swirl the plate/flask to ensure uniform distribution of the transfection complex. Incubate plate/flask in a tissue culture incubator at 37°C and 5% CO_2_ concentration.
4. ***Alternatives:*** Ca_3_(PO_4_)_2_ transfection.
  a. Prepare 293T cells for transfection as described in day 0 but incubate the plate/flask for only 4–5 h in tissue culture incubator at 37°C and 5% CO_2_ concentration to allow the cells to attach to the surface.
  b. Add chloroquine to a final concentration of 25μM, incubate for 5 min, and prepare the Ca_3_(PO4)_2_ transfection mixture. A typical template for preparing the transfection mixture for a T-25 flask transfection is shown in Table 2. Add dropwise the transfection mixture to the cells with gentle swirling.

**Table 1:** A Template for Effectene-Mediated Transfection in a 6-Well Plate

Reagent	Stock Concentration	Plasmid Ratio	Plasmid Amount	Stock Dilution	Volume
Env-expressing plasmid	1250 ng/μL	1	0.04 μg	1:40	1.28 μL
pHIVec2.luc plasmid	195 ng/μL	6	0.24 μg	1	1.23 μL
psPAX2 plasmid	1466.6 ng/μL	3	0.12 μg	1:40	3.27 μL
Enhancer					3.2 μL
Buffer					91.02 μL
Effectene					10.00 μL
Medium					490 μL
Total volume					600 μL

Cells = 5 × 10^5^ cells/well (2 mL).

DNA = 0.4 μg total/well.

  c. Gently swirl the plate/flask to ensure uniform distribution of the transfection complex. Incubate plate/flask in tissue culture incubator at 37°C and 5% CO_2_ concentration.

***Note:*** We found that Ca_3_(PO4)_2_ transfection of 293T cells is more efficient 4–5 h after seeding the cells in comparison with 293T cells that were grown overnight (14–20 h) prior to transfection.

***Note:*** According to our institutional biosafety approved protocol, at this point we transfer the plate/flask to our BSL2+ facility. Work at the BSL2+ facility follows BSL3 practices in a BSL-2 environment. The standard personal protective equipment includes disposable gown, face mask, protective shatterproof eyeglasses or face shield, double gloves, sleeve cover, and protective shoe covers. Liquid waste is decontaminated with 10% bleach and all waste is autoclaved on site.

5. Day 2: 14 h post Ca_3_(PO_4_)_2_ transfection replace the medium with a fresh DMEM medium.

**CRITICAL:** Chloroquine is toxic to cells; medium must be changed 14–16 h post transfection.

6. Day 3: Gently collect the PV-containing supernatant and centrifuge at 600–900 × *g* for 5 min at 4°C to remove cell debris. Collect the supernatant and aliquot in required volume for single-use experiments in several 1.5 mL microcentrifuge tubes. One tube should be reserved for a p24 measurement (next section).

**Table 2:** A Template for Ca_3_(PO_4_)_2_-Mediated Transfection in a T-25 Flask

Reagent	Stock Concentration	Plasmid Ratio	Plasmid Amount	Stock Dilution	Volume
Env-expressing plasmid	1250 ng/μL	1	0.9 μg	1:10	7.20 μL
pHIVec2.luc plasmid	195 ng/μL	6	5.4 μg	1	27.7 μL
psPAX2 plasmid	1466.6 ng/μL	3	2.7 μg	1	1.8 μL
ddH_2_O					182.3 μL
CaCl_2_ 2M					31 μL
HEPES buffer X 2^a^					250 μL
Total volume					500 μL

Cells = 3 × 10^6^ cells/flask (5 mL).

DNA = 9 μg total.

a Ca_3_(PO_4_)_2_ precipitates quickly. HEPES buffer should be added fast and the solution should be mixed immediately.

Alternatively, PV-containing supernatant can be filtered through a 0.45 μm filter. The two alternatives (centrifugation and filtration) may be compared for specific Envs to achieve high infectivity. Some studies have used a centrifugation step followed by 0.45 μm filtration of the PV-containing supernatant.

7. Store PV-containing tubes at −80°C for future use. PV infectivity depends on the specific HIV-1 Env used and is typically decreased ∼90% with a freeze-thaw cycle. Thus, viruses pseudotyped with Envs that intrinsically exhibit very low entry activity cannot be frozen and have to be used immediately.

**CRITICAL:** PVs should be collected with minimal mixing or minimal use of any mechanical force to preserve virus infectivity.

8. After collecting the supernatant, lyse the remaining cells in the wells/flask with luciferase lysis buffer. Transfer a few microliters of lysed cells (usually 1:10 or 1:100 of the lysate) to a 96-well white luminometer plate and measure luciferase activity. The readout reflects the efficiency of pHIVec2.luc transfection and should be close to saturation. We typically measure >2 x 10^9^ relative light unit for 2-s measurements for such cell lysates.

***Note:*** If the transfection efficiency is high, the cell lysate readout may be saturated even at 1:100 dilution. Transfection efficiency determines the relative PV titer and p24 concentration in the sample.

9. Day 3 (or 4): Each HIV-1 virion (particle) contains approximately 3000 copies of p24 protein. Thus, after viruses are disrupted to release the p24 protein, the HIV-1 p24 concentration in a specific preparation is related to the number of virions in the sample. The concentration of HIV-1 p24 protein can be measured using commercial or in-house enzyme linked immunosorbent assay (ELISA) based on HIV-1 p24 antigen capture. An efficient and economical in-house assay has been previously described (Wehrly and Chesebro, 1997); commercial p24 ELISA kits (e.g., ABL p24 capture assay) are usually more expensive, are provided in adjustable 8-well strips, and typically detect p24 concentrations at the range between 5 and 100 pg/mL in a test sample. Here we describe the use of the duo ELISA set from R&D, which provides only main components of the p24 ELISA system and represents a method of high performance at an attractive price. Using this set, we reproducibly measure p24 concentrations within a range of 31 to 500 pg/mL.
10. Duo p24 ELISA protocol:
  a. Add equal volume of PV sample to a tube containing lysis buffer (1% Triton X-100 in PBS). The lysed sample can be stored at −20°C until use.
  b. Immobilize a capture antibody (HIV Gag p24 Capture Antibody, Part 844721; Cat# DY7360-05; R&D systems) on high-binding 96-well plate (Greiner bio-one, Cat# 655061) by adding 0.4 μg of antibody in 100 μL PBS (4 μg/mL) to each well. Seal the plate and incubate overnight (14–20 h) at RT (20°C–25°C).
  c. The following day wash the wells as follows.

Wash Step: aspirate the solution from each well using a vacuum system and wash three times with 300 μL of wash buffer by adding the buffer, incubating for 30 s, and aspirating the solution. Invert the plate on a paper towel to remove remaining traces of wash buffer.

  d. Add 300 μL of blocking buffer to each well and incubate the plate at RT (20°C–25°C) for 1–2 h. Repeat Wash Step.
  e. Add 100 μL/well of p24 protein standards (HIV Gag p24 Standard, Part 844723; Cat# DY7360-05; R&D systems) at the following concentrations (serial 1:2 dilutions): 500, 250, 125, 62. 5, 31. 25, 15.625, 7.8125, and 0 (blank) pg/mL in duplicates. The measurements of these wells will be used to generate a standard curve. In addition, add 100 μL/well of test samples to respective wells. We typically test 2–3 dilutions of each sample to ensure that the measurements are within the range of the standard curve. Incubate the plate at RT (20°C–25°C) for 2 h and then repeat the Wash Step.
  f. Add 100 μL/well of detection antibody (HIV-1 Gag p24 Detection Antibody, Part 844722; Cat# DY7360-05; R&D systems) at a final concentration of 125 ng/mL and incubate at RT (20°C–25°C) for 1 h. At the end of incubation repeat the Wash Step.
  g. Add 100 μL/well of 1:40 dilution of streptavidin-HRP solution (Part 893975; Cat# DY7360-05; R&D systems) and incubate the plate at RT (20°C–25°C) for 20–30 min. At the end of incubation repeat the Wash Step.
  h. Add 100 μL/well of TMB substrate solution and incubate at RT (20°C–25°C) for 15–20 min followed by the addition of 50 μL/well stop solution. Shaking will improve the distribution of color development in the well. Within 5–30 min, measure the optical density in each well at wavelength of 450 nm. Protect the plate from light during this step. We usually cover the plate with an aluminum foil immediately after the addition of the TMB substrate.
  i. Analyze the data. Measurements are averaged and the 0 (blank) readout is subtracted from each. Averaged and blank-subtracted p24 measurements are then plotted against each p24 concentration and a standard curve is generated by fitting the measurements to a linear curve using available processing program (e.g., Excel, GraphPad). p24 concentration in a tested sample is calculated using the equation describing the fitted linear curve. R^2^ values are typically > 0.97.

**CRITICAL:** According to our institutional biosafety approved protocol, once the PV is lysed we transfer the plate/flask to a BSL2 laboratory. Liquid waste is decontaminated by 10% bleach for 20 min.

***Note:*** Another way to compare PVs is to measure the level of Env expression on the surface of each PV, which can be done by analyzing the proteins in PV preparation by western blot.

### Virus Titration

**Timing: 3–4 days**

To compare the properties of different Envs or different preparation of the same Envs, equivalent amounts of PVs are used according to (1) number of virions, which is estimated by p24 concentrations of the preparation (as described in the previous section), or (2) viral infectivity levels of Env-displaying PVs. We next describe how to titer PVs on target cells; the infectivity values measured can be used to generate a dose-response curve and to estimate the volume of each PV preparation that is required for desired infectivity level.

**Figure 3:**
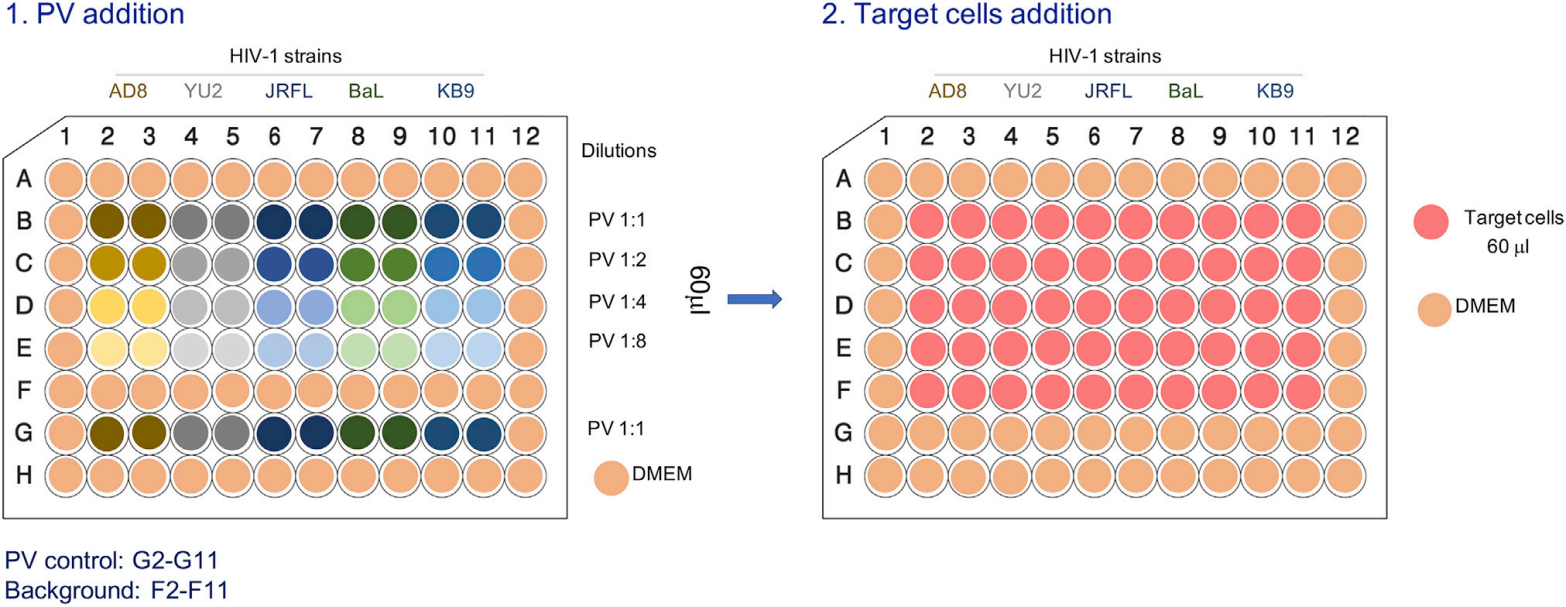
A Typical Layout of a Plate for PV Titration

11. Day 1: Prepare serial 2-fold dilutions of supernatant-containing PVs in DMEM (i.e., 1:2, 1:4, 1:8, …). PVs are very sensitive to mechanical forces and mixing should be gentle and minimal to allow even distribution of PVs in the solution without decreasing viral infectivity.
12. Add 30 μL/well of diluted PVs in duplicates or triplicates to a 96-well plate. We compared several 96-well plates and, in our hands, Greiner bio-one 96-well plates (Cat# 655083) give low background and high signal. We usually use internal wells for viral assay and add 120 μL of DMEM to otherwise empty wells to prevent evaporation of liquid in the assay wells (edge effect). An example of a typical template is shown in Figure 3.

13. Add approximately 0.5 mL of StemPro Accutase to a T75 flask containing Cf2Th-CD4/CCR5 target cells and incubate at RT (20°C–25°C) until cells are completely detached. Target cells should be grown no more than 2 days prior to the assay to ensure healthy and exponentially growing cells. StemPro Accutase is a gentle detachment agent and preserves the receptor integrity on the cell surface. Add 10 mL DMEM, centrifuge the cells at 50–100 × *g* and 4°C for 5 min, remove the supernatant, and resuspend the target cells in DMEM at concentration of 1.2 × 10^5^ cells/mL.
14. Add 60 μL/well of cells (7200 cells/well) to the wells containing PVs from step 12. Control wells should include: target cells with no PVs and PVs with no cells in the same total volume as all other wells (90 μL/well). Incubate the plate in a tissue culture incubator at 37°C and 5% CO_2_ concentration.

***Alternatives:*** Target cells can be added to a 96-well plate (7200 cells/well) one day prior to the viral assay followed by the addition of viruses on the next day.

**CRITICAL:** We found that adding target cells immediately after PV addition results in significantly higher assay readout compared with seeding target cells one day prior to assay.

***Note:*** Infection of Cf2Th-CD4/CCR5 target cells with PVs typically results in higher assay readout than the readout of infection of TZM-bl target cells. But TZM-bl cells have been extensively used in numerous reported studies. These cells contain a stably integrated copy of the *firefly luciferase* gene under the control of HIV long terminal repeat promoter in their genome, and exhibits relatively high background. TZM-bl cells are contaminated with ecotropic murine leukemia virus but this contamination does not significantly affect their performance as target cells (Platt et al., 2009). The addition of DEAE-Dextran increases the efficiency of viral entry.

15. Day 3 (or day 4): PV entry into target cells can be measured after 48 or 72 h. Measurements 72 h post infection usually results in higher readout than the measurements after 48 h, most probably because the firefly luciferase enzyme is accumulating in the target cells during this time. However, 48-h incubation typically results in high signal-to-noise ratio and in many cases is preferred due to time saving.
  a. Prepare sufficient luciferase assay buffer to measure the luciferase activity in the assay wells (100 μL/well). Thaw the luciferin substrate solution at RT (20°C–25°C); thawing can be accelerated by immersing the tube in a beaker filled with water at RT (20°C–25°C).
  b. Gently aspirate the medium from the wells of the 96-well assay plate using a vacuum system. Add 30 μL/well of luciferase lysis buffer to lyse the cells and measure the activity of luciferase using a luminometer plate reader equipped with two injectors. In our hands, Berthold luminometer provides robust and reproducible readings. After priming the injectors with the assay buffer (injector 1) and the luciferin substrate solution (injector 2), one hundred microliters of assay buffer are injected to each well, followed by a 1-s delay and a 50-μL injection of luciferin substrate solution. Light units are measured and integrated over between 2 and 10 s.

**CRITICAL:** Luciferin solution is light sensitive; keep solution protected from light at all times.

***Note:*** All luciferase reagents and the sample plate should be allowed to equilibrate to RT (20°C–25°C) before measuring luminescence as temperature variations could affect the assay readout.

### Effects of HIV-1 Env Ligands on PV Infectivity

**Timing: 3–4 days**

To measure the effect of ligands on specific Env function, PVs displaying specific Envs are incubated with increasing concentrations of the tested ligand followed by the addition of target cells to each well to measure infectivity (Herschhorn et al., 2014). Wells of infected cells without a ligand are used as reference control (100% infection) and wells with cells only are used as background control (0% infection). Additional control wells contain PV without cells. The readout for each concentration of the ligand is normalized to the two control values.

**Figure 4:**
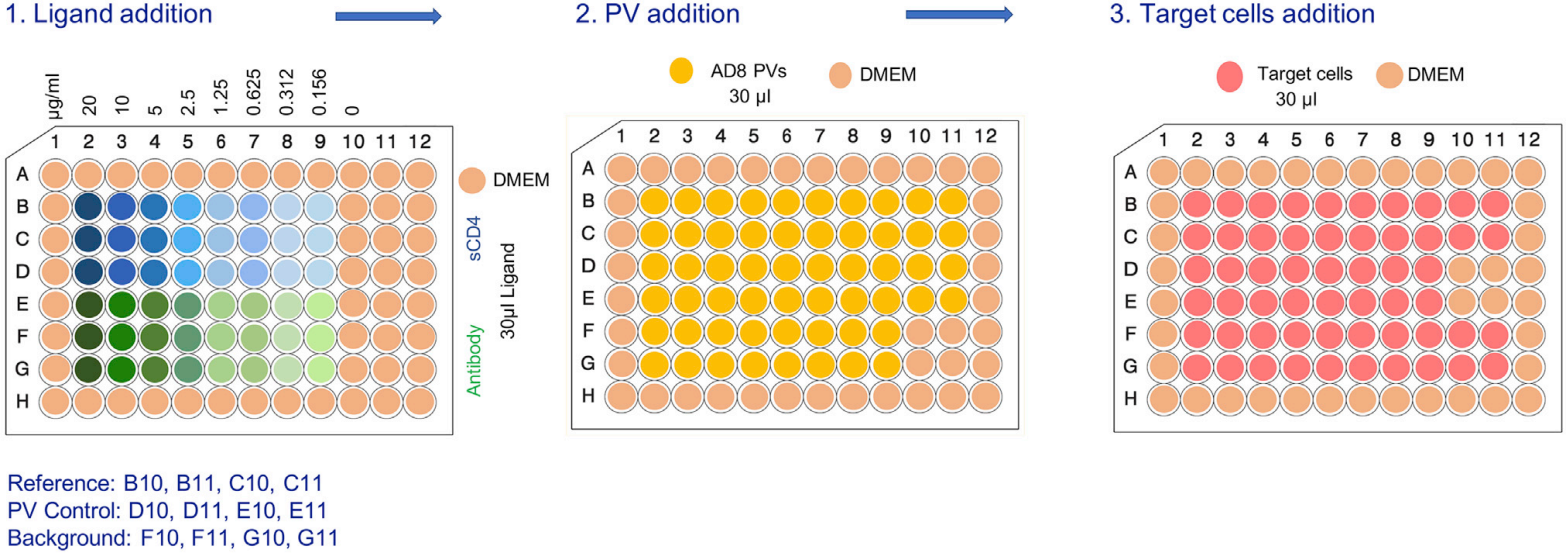
A Typical Layout of 96-Well Plate for Testing Ligand Effects on PV Infectivity

16. Day 1: Serially dilute the tested ligand in DMEM. Calculate the amount and volume needed for duplicate or triplicate measurements and dilute the ligand in microcentrifuge tubes. For small molecules dissolved in DMSO, dilute the compounds in DMSO and then dilute further to final concentration in DMEM while keeping identical DMSO concentration for all wells. Up to 2% DMSO can be used in the viral assay without any cytotoxic effects to Cf2Th-CD4/CCR5 target cells. DMSO must be included in the reference infection. An example of template is shown in Figure 4.

17. Add 30 μL of ligand at specific concentration to wells of a 96-well luminometer plate (Greiner bio-one 96-well plate (Cat# 655083)). Each ligand concentration is usually tested in duplicates or triplicates. Use 30 μL of the diluting medium for reference control (no ligands) and background (cells only) wells.
18. Add 30 μL PVs that were prediluted either to a defined amount of HIV-1 p24 or to specific infection level (or titer) to assay and reference control wells. We typically use a specific HIV-1 p24 amount in the range of 1–10 ng or infectivity values equivalent to about 1 million relative light units.
19. Repeat step 13 of Virus titration section but resuspend the target cells in DMEM at concentration of 2.4 × 10^5^ cells/mL. Add 30 μL/well of cells to the wells containing PVs, which were preincubated with a ligand, and to reference and background control wells.
20. Add 30 μL of medium to background control wells. Incubate the plate in a tissue culture incubator at 37°C and 5% CO_2_ concentration.
21. Day 3 (or day 4): Repeat step 15 (a & b) of the previous section (Virus titration).

***Note:*** Normalizing by p24 concentration reflects equivalent number of viral particles in a sample. Normalizing by PV infectivity reflects the number of viral particles that are able to mediate similar levels of entry into target cells.

***Note:*** Measuring ligands dissolved in DMSO requires the addition of DMSO to the reference cells to account for the DMSO effect

### Cold Sensitivity

**Timing: 5–6 days**

We and others have previously shown that frequent sampling of the open conformation of HIV-1 Env is associated, in many cases, with increased Env sensitivity to exposure to cold. This Env property is studied by exposing PVs to low temperature (ice) at defined intervals followed by measuring the effect on infectivity. Because different PV-containing tubes of the same batch are thawed at different time points and kept on ice for defined time frames, care should be taken to maintain PV infectivity in each tube during virus handling.

22. Day 1: Prepare approximately 10 mL of PV displaying specific HIV-1 Envs according to the steps outlined in the first section (Production of recombinant HIV-1 PVs). Aliquot the supernatant-containing PVs into 20 or more microcentrifuge tubes and store at −80°C. Measure HIV-1 p24 in the supernatant and titer the PVs as described in the previous section (Virus titration).
23. Thaw a single-use aliquot of PV preparation by immersing the tube in a 37°C water bath for exactly 1.5 min and then gently place the tube in an ice bucket. Place the ice bucket in a refrigerator to conserve the ice as much as possible during incubation. Label the tube as day 1.
24. Repeat step 23 daily for 3 consecutive days (days 2–4) at the same interval (approximately every 24 h). On day 5, repeat step 23 but without placing the tube on ice. PVs from day 5 tube are the reference for measuring 100% infectivity.
25. Dilute each PV preparation to 4 ng p24/60 μL or to a predefined infectivity level in DMEM. Add 60 μL of each PV in triplicates or quadruplicates to wells of a 96-well plate followed by the addition of 60 μL of 1.2 × 10^5^ Cf2Th-CD4/CCR5 target cells/mL (7200 cells/well). Incubate the plate in a tissue culture incubator at 37°C and 5% CO_2_ concentration.
26. Day 3 (or day 4): Repeat step 15 (a & b) of the previous section (Virus titration).

### Cell-Cell Fusion Assay

**Timing: 3–4 days**

Cell-cell fusion assay measures the fusion activity of specific HIV-1 Envs that are tested (Herschhorn et al., 2011). Effector cells are transfected with HIV-1 Tat- and HIV-1 Env-expression plasmids. TZM-bl cells serve as target cells. They express the CD4/CCR5 receptors and contain a stably integrated copy of the *firefly luciferase* gene under the control of HIV long terminal repeat promoter in their genome. Fusion of effector and target cells during co-culture leads to diffusion of HIV-1 Tat to the target cells and activation of firefly luciferase transcription.

**Figure 5:**
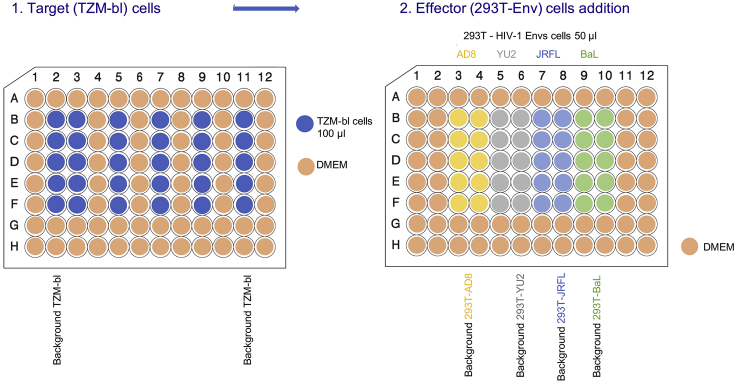
A Typical Layout of a Plate for Cell-Cell Fusion Assay

27. Day 1: Follow the steps outlined in the first section for Ca_3_(PO_4_)_2_ transfection (step 4) to transfect 293T cells with HIV-1 tat- and HIV-1 Env-expression plasmids at a ratio of 1:6 in a 6-well plate (Table 3). Incubate transfected cells in a tissue culture incubator at 37°C and 5% CO_2_ concentration.

28. Day 2: Detach TZM-bl cells and centrifuge the cells at 50–100 × *g* and 4°C for 5 min. Resuspend the cells in DMEM, count the cells and adjust the concentration of 1 × 10^5^ cells/mL. Add 100 μL of cells to designated wells of a 96-well plate (Figure 5).
29. In addition to assay wells containing co-culture of effector (293T) and target (TZM-bl) cells, wells containing only effector cells or only target cells are used as control. We usually measure fusion after 2, 4, and 6 h; each time point requires a separate plate. See an example layout in Figure 5. Incubate the plate overnight (16–20 h) in a tissue culture incubator at 37°C and 5% CO_2_ concentration.
30. Day 3: Detach the transfected 293T cells using 5 mM EDTA in PBS and transfer to a 15-mL centrifuge tube. Add 10 mL DMEM and centrifuge cells at 50–100 × *g* and 10°C for 5 min. Resuspend the transfected 293T cells in fresh medium at concentration of 2 × 10^5^ cells/mL.
31. Remove 50 μL from assay wells that are designated for co-culture and contain TZM-bl target cells, and add 50 μL of transfected 293T cells (10,000 cells/well). Repeat this step for empty wells that contain DMEM medium and are designated as control. Keep wells with TZM-bl cells only as second control (Figure 5).

**Table 3:** A Template for Transfecting 293T Cells in 6-Well Plate for a Cell-Cell Fusion Experiment

Reagent	Stock Concentration	Plasmid Ratio	Plasmid Amount	Stock Dilution	Volume
Env-expressing plasmid (e.g., pAD8-M)	1250 ng/μL	6	1.8 μg	1:10	14.4 μL
pTat plasmid	70 ng/μL	1	0.3 μg	1	4.29 μL
ddH_2_O					91.31 μL
CaCl_2_ 2M					15 μL
HEPES buffer X 2^a^					125 μL
Total volume					250 μL

Cells = 5 × 10^5^ cells/well (2 mL).

DNA = 2.1 μg total.

a Ca_3_(PO_4_)_2_ precipitates quickly. HEPES buffer should be added fast and the mixture should be mixed immediately.

32. Incubate the plates in a tissue culture incubator at 37°C and 5% CO_2_ concentration and measure the fusion activity after 2, 4, and 6 h (1 plate for each time point) by following step 15 (a & b) of the Virus titration section.

## Expected Outcomes

Our protocol provides molecular tools to study the function of HIV Envs. We usually produce high titer PVs from one T-25 flask or 6-well plate of transfected 293T cells (3 million cells/flask; 0.5 million/well of 6-well plate) using both Ca_3_(PO_4_)_2_ as well as commercial Effectene transfection reagent. Based on luciferase expression system, signal-to-noise ratio for highly efficient HIV-1 Envs (e.g., HIV-1_JRFL_ Envs, see below) should be > 1000. The experiments should give the following information for each specific Envs:

1. Sensitivity to ligands (e.g., antibodies, sCD4). This is usually calculated as IC_50_ (see below).
2. Fusion ability. Comparison of the fusion activity between different Envs.
3. Sensitivity to cold. Envs of many primary HIV-1 strains are not sensitive to cold exposure up to at least 96 h. This property usually correlates with a more closed conformation. Nevertheless, some amino acid changes and Envs of some strains are more open than others and are expected to have high sensitivity to cold.

Integration of the above Env properties will provide a comprehensive view on HIV-1 Env function and a basis for comparison between Envs of different strains as well as a framework to study the effect of amino acid changes on HIV-1 Env activity.

## Quantification and Statistical Analysis

**Figure 6:**
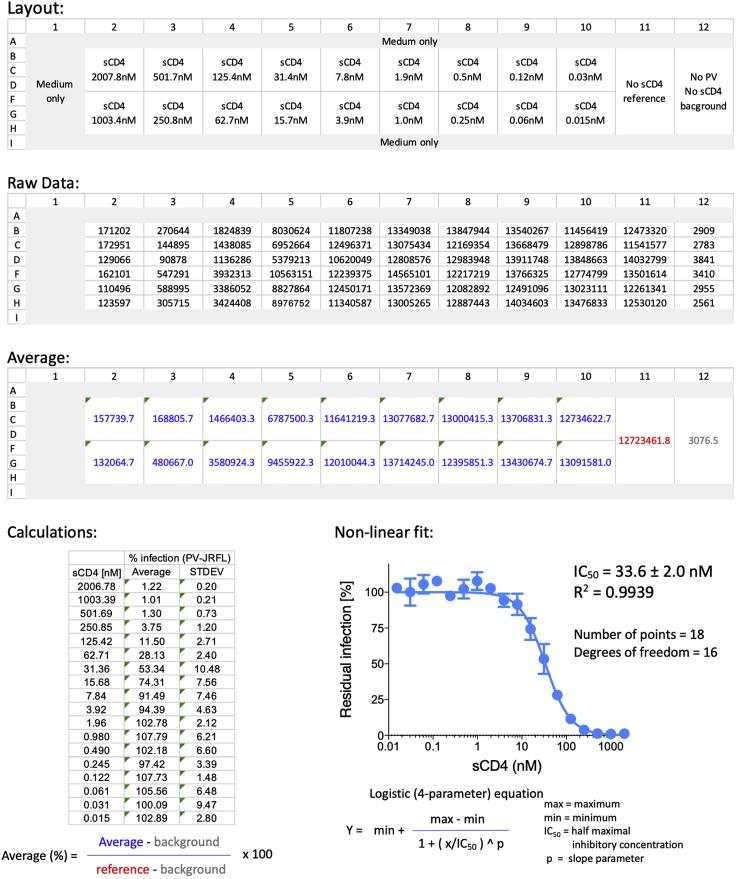
A Workflow for IC_50_ Calculation A step-by-step analysis of dose-response data generated measuring the effect of different concentrations of sCD4 on HIV-1_JRFL_ pseudovirus infection. We typically calculate the average values using an Excel spreadsheet and then export the data to Prism (GraphPad). We fit the data to the logistic equation after adding the equation to the program (Herschhorn et al., 2008, 2010). For typical dose-response curves, the following constrains are used for consistency: max value = 100%; min value = 0%; p < 3. Ligands that either enhance infection or do not completely inhibit infections should be treated individually and all parameters (Max, Min, IC_50_, and p) should be reported.

The detailed example in Figure 6 provides a step-by-step guidance for calculating half maximal inhibition concentration (IC_50_) of HIV_JRFL_ Envs by soluble CD4. The assay was performed in triplicates in a 96-well plate according to our protocol. Results are adapted from Harris et al. Cell Reports 2020.

## Limitations

• Our protocol cannot measure HIV-1 Env function of Envs with very low fusion activity. Some primary HIV-1 strains can replicate in cells but they enter target cells with very low efficiency. As a result, we will not be able to detect the viral entry mediated by of some Envs in our system. For example, in our hands a codon-optimized version of BG505 showed a very low infectivity and could not been used for accurate and reliable measurements. Other Envs do not tolerate freeze-thaw cycle and have to be assayed immediately after PV preparation. In this case, p24 is measured immediately after PV preparation and the virus is then used. Alternatively, several dilutions can be used for the viral assay immediately after preparation, and p24 concentration of each dilution is determined at a later time point. The latter approach does not allow to use predefined p24 concentration but a dilution with desired p24 concentration can be selected for analysis.
• PV infection follows Poisson distribution and low levels of infection usually result in high variability between the readout from different wells on the same plate and between the measurements of independent experiments. Thus, reliable results require Envs that can mediate reasonable level of infection or can be concentrated without significant loss of activity. PVs can be concentrated by ultracentrifugation, membrane-based anion exchange chromatography or precipitation using polyethylene glycol 6000 (Kutner et al., 2009; Reiser, 2000).

## Troubleshooting

### Problem 1

Low infectivity of PVs (steps 1–7).

### Potential Solution

• Test the luciferase assay reagents: luciferin substrate, assay buffer, DTT and ATP; and prepare new reagents if needed.
• Make sure 293T cells are healthy. If needed, thaw a new tube of 293T cells.
• Test transfection reagents with a reporter protein
• Prepare PVs with efficient Envs (e.g., HIV-1_JRFL_ Envs) to rule out technical problems during PV preparations
• Use PVs without freezing. Envs of some HIV-1 strains are poor and sensitive to freeze-thaw cycles.
• The polycation DEAE-Dextran can be supplemented in the assay medium to increase the PV infectivity while performing TZM-bl neutralization assays (https://www.hiv.lanl.gov/content/nab-reference-strains/html/Protocol-for-Preparation-and-Titration-of-HIV-1-Env-pseudotyped-Viruses_Apr-2020.pdf). However, including DEAE-Dextran in the assay can result in up to 3-fold change in the neutralizing antibody activity (Sarzotti-Kelsoe, M et al., 2014). Thus, if DEAE-Dextran is used in experiments that compare different viruses it should be used for all samples tested.

### Problem 2

High variation of measurements (steps 11–15).

### Potential Solution

• Use higher titer of PV. In our hands, infection of Cf2-CD4/CCR5 cells for 48 h that leads to approximately 1 × 10^6^ relative light units, which are measured during 2-s integration on the Centro LB 960 XS^3^ Berthold luminometer, gives reliable and reproducible readouts.
• Incubate PV-infected target cells for 72 h. Readout are usually higher and more uniform after a 72-h incubation
• Make sure Luciferin substrate is at RT (20°C–25°C) during the assay measurements

### Problem 3

Cell aggregates when preparing 293T cells for cell-cell fusion (step 27).

### Potential Solution

• Gently mix with serological pipette to break cell aggregates
• Filter the cells and then count cell number

## Resource Availability

### Lead Contact

Further information and requests for resources and reagents should be directed to and will be fulfilled by the Lead Contact, Alon Herschhorn (aherschh@umn.edu).

### Materials Availability

Materials generated in this study are available upon request.

### Data and Code Availability

This study did not generate any unique datasets or code.
